# Initial-onset acute and chronic recurrent stages are two distinctive courses of Vogt-Koyanagi-Harada disease

**DOI:** 10.1186/s12348-020-00214-2

**Published:** 2020-09-14

**Authors:** Cristhian A. Urzua, Carl Herbort, Rodrigo A. Valenzuela, Ahmed M. Abu El-Asrar, Lourdes Arellanes-Garcia, Ariel Schlaen, Joyce Yamamoto, Carlos Pavesio

**Affiliations:** 1 Department of Ophthalmology, Faculty of Medicine, University of Chile, Independencia, 1027 Santiago, Chile; 2 Laboratory of Ocular and Systemic Autoimmune Diseases, Faculty of Medicine, University of Chile, Santiago, Chile; 3 Facultad de Medicina, Clinica Alemana-Universidad del Desarrollo, Santiago, Chile; 4 Retinal and Inflammatory Eye Diseases, Centre for Ophthalmic Specialized Care (COS), Clinic Montchoisi Teaching Centre, Lausanne, Switzerland; 5 Department of Ophthalmology, University of Lausanne, Lausanne, Switzerland; 6 Departamento de Ciencias Quimicas y Biologicas, Facultad de Salud, Universidad Bernardo O Higgins, Santiago, Chile; 7 Department of Ophthalmology, Dr. Nasser Al-Rashid Research Chair in Ophthalmology, College of Medicine, King Saud University, Riyadh, Saudi Arabia; 8 Inflammatory Eye Diseases Clinic, “Dr. Luis Sanchez Bulnes” Hospital Asociación para Evitar la Ceguera en México (APEC), Mexico DF, Mexico; 9 Hospital Universitario Austral, Hospital de Clinicas de Buenos Aires, Buenos Aires, Argentina; 10 Department of Ophthalmology, Faculdade de Medicina FMUSP, Universidade de Sao Paulo, Sao Paulo, Brazil; 11 National Institute for Health Research, Biomedical Research Centre at Moorfields Eye Hospital, NHS Foundation Trust and UCL, Institute of Ophthalmology, London, UK

**Keywords:** Vogt-Koyanagi-Harada disease, Initial-onset acute VKH, Chronic VKH, Categorization

## Abstract

**Purpose:**

To describe distinctive stages of Vogt-Koyanagi-Harada (VKH) disease: initial-onset acute versus chronic recurrent disease.

**Methods:**

A comprehensive literature review regarding stages and clinical presentations of VKH disease was conducted.

**Results:**

Despite a list of signs that has been described as characteristic features of early or late phases of VKH disease, the current classification -developed by an international committee and published in 2001- does not consider a distinction regarding the time from onset of disease symptoms, and specific findings observed at certain time point from the symptoms presentation and outcomes related to the stage of VKH disease. In that sense, chronic recurrent VKH disease is more refractory to treatment and is associated with a higher rate of complications. Accordingly, this subset of VKH patients has poorer functional and anatomical outcomes than patients with an initial-onset acute disease.

**Conclusions:**

An early clear distinction of VKH phenotype [Initial-onset acute versus chronic recurrent disease] should be considered in each clinical scenario, evaluating the delay in diagnosis and the clinical presentation, since it may help clinicians to perform a correct disease prognosis categorization and thus to make treatment decisions in terms of potential refractoriness or expected clinical outcomes.

## Introduction

Vogt-Koyanagi-Harada (VKH) disease is an inflammatory disorder that presents bilateral intraocular inflammation, associated with exudative retinal detachments, and systemic manifestations in the auditory, integumentary and central nervous system (CNS) [1, 2].

The frequency of VKH disease in the world is variable. It appears to be more prevalent in people with Asian, Hispanic, Indian, Native American, or Mediterranean origin, accounting for 7%–22.4% of uveitis referrals [3–6].

## Clinical features

The primary ocular pathological feature of VKH disease is a diffuse thickening of the uveal tract caused by a non-necrotizing granulomatous inflammation. This thickening is more prominent in the posterior part of the uvea, the juxtapapillary choroid. In the acute phase of VKH disease, exudative retinal detachment with collection of subretinal fluid most likely results from alterations in the retinal pigment epithelium (RPE) as “upstream” effect of choroidal compromise [7]. In addition, the initial stage of disease presents with optic disc hyperaemia and swelling, subsequently involving the anterior segment (AS) and finally developing into chronic recurrent granulomatous anterior uveitis. If not properly treated, relentless subclinical choroidal inflammation may be involved in the pathogenesis of progressive posterior segment depigmentation resulting in “sunset glow fundus” appearance and chorioretinal atrophy [5, 8–12].

## Diagnosis

The diagnosis is based on clinical presentation and ancillary testing, with a set of diagnostic criteria developed by an international committee on VKH disease, published in 2001 [2, 13]. Using this approach, patients can be classified as having probable (only ocular findings), incomplete (ocular plus integumentary or neurological findings) or complete VKH disease (ocular plus integumentary plus neurological findings) [2, 5, 14].

The diagnosis of VKH disease is made by clinical aspects, and thus its diagnosis can be a challenge, due to the lack of a single laboratory test or pathognomonic feature to distinguish a VKH patient among the whole group of patients with uveitis. In this regard, despite the above-mentioned revised diagnostic criteria, Standardization of Uveitis Nomenclature (SUN) group has recently reported only an overall moderate agreement among uveitis experts for the diagnosis of VKH disease (mean kappa 0.4) [15].

In the diagnostic criteria consensus, a list of signs was described as characteristic features of early or late phases of the condition. However, this classification did not consider time from the onset of symptoms, and specific findings observed at certain time point from the symptoms presentation and outcomes related to the stage of VKH disease were not introduced [13, 16]. Moreover, as an attempt to introduce a categorization of VKH patients in clinical studies, different intervals of time have been used [5, 14, 17–20]. Another major shortcoming of these revised criteria is the fact that highly sensitive diagnostic modalities, such as indocyanine green angiography (ICGA) [21], measurement of choroidal thickness using enhanced depth imaging optical coherence tomography (EDI-OCT) [22] or swept source optical coherence tomography (SS-OCT) [23], and high-frequency ultrasound biomicroscopy (UBM) for in vivo visualization of the anterior segment [24], especially crucial for early identification of the inflammatory process, diagnosis of atypical cases and follow up of the disease, are missing.

In that regard, since prognosis is significantly affected by the stage of the disease and the presence of subclinical inflammation [5, 8–12], the use of a more comprehensive classification that considers ancillary testing and a clear distinction between initial-onset acute and chronic recurrent disease courses, is advisable. The poor response to therapy, among patients with a chronic recurrent granulomatous AS inflammation has been described [20, 25]. Moreover, this subset of patients has more complications [8, 26] and a trend to have more severe ocular and systemic manifestations of the disease. El Asrar et al. found a higher frequency of posterior synechiae and anterior chamber cells at diagnosis in patients who later turned to have this chronic condition [8, 26].

## VKH disease courses

### Characteristics of initial-onset acute VKH disease

The very early stage of the disease is characterized by prodromal symptoms with neurological and auditory manifestations such as headache, malaise and meningismus. Patients complain of periorbital pain in more than a third of cases, and occasionally photophobia with tearing may follow within 1–2 days. The auditory disturbances include a dysacusis that is often not perceived by the patient and, occasionally, mild vestibular syndrome with vertigo [27]. Importantly, these neurological and auditory manifestations are not presented in all VKH cases, and even a significant portion of patients may have ocular signs without the prodromal phase of the disease [1, 7, 14, 25].

Several days after the appearance of the prodromal phase, the acute phase of the disease begins, characterized by acute uveitis, with diffuse granulomatous choroiditis, with secondary exudative retinal detachments and optic disc hyperaemia and swelling [1, 27, 28].

### Characteristics of chronic recurrent VKH disease

The chronic recurrent course of VKH disease must be distinguished from initial-onset acute course. For too long this difference has not been emphasized resulting in inadequate understanding ot the disease and in insufficient treatment. It is characterized by recurrent granulomatous AS inflammation with “sunset glow fundus” and peripheral scars. These scars are areas of focal chorioretinal atrophy with loss of retinal pigment epithelium [1, 7]. Vision-threatening complications have clearly been recognized to occur in the chronic recurrent phase of VKH disease, namely cataract, glaucoma, choroidal neovascular membranes, subretinal fibrosis, chorioretinal atrophy and sunset glow fundus [29]. The occurrence of these complications is known to be associated with a worse visual outcome [30] and mean retinal sensitivity [26]. Even after early corticosteroid monotherapy treatment of initial-onset disease, a significant proportion of cases goes to chronic disease, developing chronic recurrent granulomatous inflammation and progressive depigmentation of the fundus, resulting in sunset glow fundus appearance [31].

Several studies have reported the significant association between the incidence of chronic ocular inflammation and the development of sunset glow fundus [26, 32, 33]. In this regard, even in the absence of manifest inflammation of posterior segment, ICGA studies have shown progression to sunset glow fundus as a consequence of occult inadequately controlled choroidal inflammation [11], and subclinical choroidal inflammation during AS recurrences [9].

In addition, histopathologic analyses of eyes with “sunset glow fundus” in patients with VKH disease have revealed the presence of scattered inflammatory infiltrate of predominantly T-lymphocytes in the thickened choroid with notable disappearance of choroidal melanocytes [10]. These findings suggest that ongoing subclinical choroidal inflammation due to inadequate immunosuppression is involved in the pathogenesis of progressive posterior segment depigmentation resulting in “sunset glow fundus” appearance and chorioretinal atrophy [9, 10]. In the majority of these cases, treatment should be prolonged and/or restarted for each recurrence. However, efficacious therapy able to stop the disease at this stage has not been found yet.

## Different definitions of initial-onset acute and chronic recurrent VKH

Working groups on VKH disease have published diverse terminologies and definitions for disease classification. However, up till now there has been no agreement neither in the terminology nor in the development of clear and explicit definitions for the different VKH courses described here. In this regard, patients with an early course of the condition at diagnosis have been classified as having an early [15], a new onset-acute [19], an acute-resolved [31], an acute VKH disease [18] or an initial-onset acute VKH disease [8, 17]. On the other hand, subjects with late presentation have been referred to as subacute [2, 34], chronic [17, 26, 35] or late [7, 15, 16].

Different approaches may be used to classify the stage of this disorder. The time frame from the clinical onset of the disorder has been extensively used for this purpose, and authors have considered symptoms presentation and timing of specialist evaluation in order to formulate a proper diagnosis.

In that sense, different intervals of time have been used. For example, Arellanes’s group considered 2 weeks or less as an evolution time for defining initial-onset acute VKH patients [18]. This distinction was based in Chee et al. findings, who observed that an early treatment (within 2 weeks from onset of symptoms) of the disease with high doses of corticosteroids, led more frequently to complete resolution and fewer complications than the administration of low or even high but late (between 2 and 4 weeks from onset of symptoms) doses of oral corticosteroids [36]. Others authors have considered a longer interval of time between symptoms onset and diagnosis: 1 month [14, 17], 2 months [19] and 3 months [25]. Da Silva et al. classified VKH disease as early when symptoms of the disease were present for less than 4 weeks. This classification was in agreement with their findings of classic uveitic phase manifestations only in the early VKH group [14]. Abu el Asrar et al [17] considered those cases of VKH presenting with granulomatous choroiditis, exudative retinal detachment, and disc hyperaemia and swelling, as initial-onset acute disease, in accordance to the findings of Yang et al. [5] They observed that those patients who fulfilled this category presented to their consultation in a range of time from the onset of symptoms between 1 to 30 days [17]. Nakayama et al. excluded from their study those patients presenting with sunset glow fundus, or those who presented to their consultation with more than 2 months from the onset of VKH disease symptoms [19]. Urzua et al. found that in those VKH patients with poor response to corticosteroids, immunomodulatory treatment was successful in improving visual function when it was initiated at an average time of 3 months [25]. In their retrospective cohort study, poor response to corticosteroid was associated with fundus depigmentation and chronic disease, among other prognostic factors [25]. Yang et al. separated their VKH cohort in three groups: less than 2 weeks at diagnosis, between 2 weeks and 2 months, and patients with a consultation after 2 months of symptoms onset [5]. This study addressed the dynamic clinical features of VKH disease characterized by early posterior uveitis and, if not properly controlled, followed by a subsequent recurrent granulomatous uveitis [5]. However, the intermediate group (between 2 weeks and 2 months) appears redundant because it shown mostly clinical features described here for initial onset acute VKH, except in those with “sunset glow fundus” which is characteristic of chronic stage.

On the other hand, as well as temporal characterization of the disorder is relevant, typical features that are found in each clinical course should be taken into account: chronic AS inflammation, exudative retinal detachment, “sunset glow fundus” or integumentary changes such as polliosis, vitiligo, and alopecia.

## Implications for clinical practice

Several other inflammatory entities -such as multiple sclerosis (MS), and juvenile idiopathic arthritis (JIA)- have specific courses of the disease, with distinctive clinical presentations associated with different anatomic and functional prognoses related to the degree of tissue damage. In such cases, an appropriate classification of the presenting phenotype guides the disease management [37, 38]. Therefore, herein we propose that, somehow, VKH disease has significant differences when first seen in the initial-onset acute or in the chronic recurrent stages. The recognition of these different courses of VKH disease carries weight because a diagnosis and proper treatment in the early stage imply a better prognosis and even a potential cure of the disease, since the main structure that generates inflammation at disease onset is the choroid and thus if inflammation is eradicated here, restoration of the blood-ocular barrier can be expected [39, 40]. Accordingly, in most reports in the literature these two different stages (initial-onset acute and chronic recurrent disease), have shown completely different behavior as far as impact of therapy is concerned [39, 40].

Recently, Abu El-Asrar analyzed their VKH disease cases. As previously reported, all patients were treated with mycophenolate mofetil in addition to systemic corticosteroids as first line therapy [17]. Patients were divided patients into three groups based on the clinical pattern at presentation: patients with initial-onset acute uveitis without AS inflammation, patients with initial-onset acute uveitis with AS inflammation and those with chronic recurrent uveitis. Data analysis demonstrated that none of the eyes that presented with initial-onset acute VKH disease without AS inflammation, developed cataract during follow-up period. On the other hand, 13.6% of eyes with initial-onset acute VKH disease with AS inflammation and 19.4% of eyes with chronic recurrent VKH disease developed cataract during follow-up period. In addition, none of the eyes that presented with initial-onset acute VKH disease without AS inflammation developed other complications including choroidal neovascular membrane, glaucoma, “sunset glow fundus” and chorioretinal atrophy during follow-up period (Abu El-Asrar, et al. unpublished data). These findings suggest that prompt treatment in early phases of initial-onset acute VKH disease without AS inflammation is mandatory to prevent the development of cataract and other complications.

The classification alluded here is beneficial in reporting studies that discuss treatment of VKH patients, allowing progression towards a new therapeutic approach for initial-onset acute and chronic VKH disease. Such classification is much more useful than the presently used classification mixing the two sub-entities, initial onset disease and chronic disease, resulting to late proper diagnosis and thus insufficient treatment at presentation [2]. This explains also why what was termed “complete VKH disease” in this system is almost never diagnosed as it mixes acute and chronic signs that do not occur concomitantly. The shortcomings of this system have already been mentioned [27]. Yang et al. described an improved set of diagnostic criteria for VKH (DCV), which represents a more comprehensive and practical method of diagnosis and classification, considering distinctive clinical features and specific findings on ancillary testing (OCT, B-scan ultrasonography, EDI-OCT, and fluorescein angiography) [16]. In this regard, a better accuracy for diagnosis was observed in as much as early and late categories were considered. In a Chinese population, authors found that the distinction of these two phases of the condition improved the accuracy of the diagnosis, with an area under the receiver operating characteristic curve (ROC) of 0.9 (versus 0.8 of the revised diagnostic criteria). Moreover, they reported better negative predictive value and sensitivity using this new approach for diagnosis [16].

Therefore, it is urgent to revise our approach to VKH disease in order to improve the diagnostic performance and to avoid chronic evolution to our patients, as we have cumulative evidence that this condition can be transformed into a less severe disease by early and adequate therapeutic intervention. In that sense, those patients presenting in a chronic recurrent stage at diagnosis should be more intensively treated and treatment maintained.

Due to the importance of avoiding the chronic course of VKH disease, it is advisable to look for potential clinical signs of inflammation, even evidence of subclinical choroiditis. Therefore, monitoring should guide the uveitis specialist to eradicate choroidal recurrence by increasing therapy each time there is significant choroidal subclinical inflammation [31]. Recent works have compared the utility of ICGA, EDI-OCT and SS-OCT during follow-up of patients with VKH disease [21–23, 41, 42]. ICGA is a technique using indocyanine green dye to visualize the choroid and delineate the choroidal circulation, giving an evaluation of inflammatory lesions in various conditions with high sensitivity [21]. On the other hand, EDI-OCT and SS-OCT are a non-invasive, more affordable technique that are used to evaluate choroidal thickness and morphology, providing a detailed and objective visualization of the choroid, and thus can be used to characterize inflammatory activity in disorders involving the posterior segment [22, 23]. In this regard, SS-OCT has been reported to be superior to EDI-OCT, presenting higher resolution and more measurable images, allowing in the same scan a precise qualitative and quantitative evaluation of retinal and choroidal changes [23, 42]. ICGA has been described as more sensitive than EDI-OCT to detect disease recurrences or subclinical inflammation in VKH patients [21]. Additionally, UBM examination may have an important role in evaluation of response to treatment in acute uveitic/recurrence stages, and in early detection of recurrences [43].

During the process of making treatment decisions, ancillary tests are very important -even if this may be time consuming- in order to complete a comprehensive evaluation and to detect possible subclinical and to classify the patient risk of inflammatory recurrences. In this regard, ICGA appears to be the first option to be carried out, with a special consideration of EDI-OCT and UBM in geographic areas where ICGA is not available.

## Conclusion

The development of uveitis in VKH disease goes through different stages [Initial-onset acute and chronic VKH disease], which are important to differentiate as the impact on prognosis and disease resolution will depend on the category of disease and how appropriately treatment was introduced. Therefore, an expert panel would be advisable in order to review the concepts discussed here. Importantly, consensus regarding terminology and disease categorization as well as a standardization of the time-frame to define each category, should be discussed. Additionally, we suggest to include ancillary tests during follow-up (i.e., ICGA, EDI-OCT or SS-OCT) in order to have a comprehensive evaluation and an early detection of an active inflammatory process.

## Data Availability

Not Applicable.

## Abbreviations

AS: Anterior segment
CNS: Central Nervous System
DCV: Diagnostic criteria for VKH
EDI-OCT: Enhanced depth imaging OCT
ICGA: Indocyanine green angiography
JIA: Juvenile idiopathic arthritis
MS: Multiple sclerosis
OCT: Optical coherence tomography
ROC: Receiver operating characteristic curve
RPE: Retinal Pigment Epithelium
SS-OCT: Swept source OCT
SUN: Standardization of Uveitis Nomenclature
UBM: Ultrasound biomicroscopy
VKH: Vogt-Koyanahi-Harada
